# The attraction of virgin female hide beetles (*Dermestes maculatus*) to cadavers by a combination of decomposition odour and male sex pheromones

**DOI:** 10.1186/1742-9994-9-18

**Published:** 2012-08-14

**Authors:** Christian von Hoermann, Joachim Ruther, Manfred Ayasse

**Affiliations:** 1 Institute of Experimental Ecology (Biology III), Ulm University, Albert-Einstein-Allee 11, Ulm, 89069, Germany; 2 Institute of Zoology, University of Regensburg, Universitätsstraße 31, Regensburg, 93053, Germany

**Keywords:** Forensic entomology, *Dermestes maculatus*, (*Z*9)-unsaturated fatty acid isopropyl esters, Cadaver odour, Pheromone, Piglet cadaver

## Abstract

**Introduction:**

The hide beetle *Dermestes maculatus* (Coleoptera: *Dermestidae*) feeds as an adult and larva on decomposing animal remains and can also be found on human corpses. Therefore, forensic entomological questions with regard to when and how the first receptive females appear on carcasses are important, as the developmental stages of their larvae can be used to calculate the post-mortem interval. To date, we know that freshly emerged males respond to the cadaver odour of post-bloated carcasses (approximately 9 days after death at T_mean_ = 27°C), being attracted by benzyl butyrate. This component occurs at its highest concentration at this stage of decay. The aim of our study was to determine the principle of attraction of virgin females to the feeding and breeding substrate. For this purpose, we tested the response of these females to headspace samples of piglet cadavers and male sex pheromones [(*Z*9)-unsaturated fatty acid isopropyl esters] in a Y-olfactometer. Because we expected that such an odour combination is of importance for virgin female attraction, we tested the following two questions:

1) Are virgin female hide beetles attracted by a combination of cadaver odour and male sex pheromones?

2) During which decomposition stage do the first virgin females respond to cadaver odour when combined with male sex pheromones?

**Results:**

We found that young virgin females were attracted to the cadaver by a combination of cadaver odour and male sex pheromones. Neither cadaver odour alone nor male sex pheromones alone was significantly more attractive than a solvent control. Our results also gave a weak indication that the first young virgin females respond as early as the post-bloating stage to its associated decomposition odour when combined with male sex pheromones.

**Conclusions:**

Our results indicate that freshly emerged males possibly respond to cadaver odour and visit carcasses before virgin females. Being attracted to cadavers when male sex pheromone is perceived as well, virgin females can optimise their reproductive possibilities.

## Introduction

The decomposition process of a vertebrate cadaver is accompanied by the entomofaunal succession of a huge richness of carrion-associated species [1]. During the first stage of decomposition, the so-called fresh stage (Table 1), the first arriving insects are members of the families Calliphoridae and Sarcophagidae [2]. Their eggs and larvae need moist tissue for successful development [3]. During the next stage, the bloated stage (Table 1), significant maggot masses can be observed [2]. In the post-bloating stage, not only large feeding masses of fly maggots, but also predatory members of the Staphylinidae and Histeridae can be observed in their role as predators of fly maggots and, at the end of this stage, most of the maggots have left their food substrate for pupation [2]. This time point is preferred by adult dermestid beetles (Table 1), because they feed on the remaining cadaver skin and ligamentous tissue [4]. In the next two stages, the advanced decay stage and the dry-remains stage, coleopteran species such as members of the Cleridae, Dermestidae (Table 1) and Scarabaeidae will dominate the fauna of the cadaver [2,5].

**Table 1:** Compilation of five different stages of decomposition in correlation with dermestid beetle occurrence

**Stage**	**Time**	**Description**	**Occurrence of dermestids**
	**[days p.m.**^**a**^**]**		
fresh	~ 0 - 2	autolytic processes	
bloated	~ 2 - 6	inflated abdomen through	
		gaseous byproducts of putrefaction	
post-bloating	~ 5 - 11	skin rupture and release of trapped	arriving of adults in late post-bloating
		putrefactive gases	
advanced	~ 10 - 25	most of the flesh has disappeared,	predominance of adults and first larvae
decay		some soft tissue remains in the	
		abdomen	
dry remains	~ > 25	only bones, hair and remains of	predominance of larvae
		dried-out skin	completion of larval development

^a^days post mortem.

Data are modified after Powers [6], Centeno et al. [7], Goff [2] and Anderson & Vanlaerhoven [8].

The volatile organic compounds (VOCs), which are released during the process of decomposition, are most likely responsible for the attraction of the above-mentioned cadaver insects [9,10], provided that the remains are sufficiently exposed. Otherwise, some restrictions occur with respect to host detection and the time required for reaching the remains when cadavers are buried, covered or wrapped [11-13]. Such scenarios, in addition to the ever present environmental parameters such as temperature, humidity and season, can influence the species composition and arrival time of the above-mentioned necrophagous fauna [8,14,15].

One of the cadaver insects that is consistently and exclusively associated with carcasses is the hide beetle *Dermestes maculatus* De Geer (Coleoptera: *Dermestidae*) [4]. More than 3000 adult hide beetles have been recorded on a single cadaver, which can be completely consumed to the bones by their necrophagous larvae. These larvae feed and remain on the dried-out cadaver skin after most of the adult beetles have departed following adult feeding and mating activities in mixed-sex aggregations on moist muscle tissue of the earlier decomposition stages [4,16].

In approximately five to seven weeks, the life cycle of the hide beetle is completed on a cadaver in the dry-remains stage or on stored animal products, provided that the environmental conditions are optimal [17] with the highest survivorship between 25°C and 30°C [18]. Between 5 and 11 larval stages are described in the literature [19,20]. A pupation site inside the cadaver or outside on wooden material will be explored by the larvae during the last ten days of the final instar [17]. The emerged adults, which are able to fly, can reach another cadaver in later post-bloating stage [2,19] where multiple mating events take place [21]. Within the first 24 h after the first mating, females start to oviposit [21]. During their lifespan of 4 to 6 months, they oviposit and remate continuously [22].

A polygamous mating strategy of *D. maculatus* has been confirmed in small mixed-sex laboratory aggregations [21]. Interestingly, males of intermediate age benefit from the presence of other males in mating aggregations because of the achievement of the highest proportion of available matings when in competition with younger and older male hide beetles [23]. Females also benefit from intermediate-aged mates with a higher success of fertilization compared with matings with younger or older conspecific males [23].

On a more chemical point of view, unsaturated esters of higher volatility elicit strong olfactory receptor responses and cause aggregation [24-26]. Only males possess a subepidermal exocrine pheromone gland with an outlet in the fourth abdominal sternite. Freshly emerged males have a group of cells that develop within a time span of about 14 days (at 25°C) into a secretory gland. During this period, the pheromone production increases gradually up to a maximal level [27]. Eleven saturated and unsaturated fatty acid isopropyl esters have been identified in the pheromone gland [25]. Among the four identified (*Z*9)-configurated isopropyl esters, isopropyl (*Z*9)-dodecenoate and isopropyl (*Z*9)-tetradecenoate elicit the highest electrophysiological and behavioural responses. Isopropyl (*Z*9)-hexadecenoate elicits lower responses and isopropyl (*Z*9)-octadecenoate is completely inactive on male antennae [28]. The behavioural responses of hide beetles have been tested in a dual choice bioassay [29]. However, only the response of unmated male hide beetles to (*Z*9)-unsaturated fatty acid isopropyl esters has been tested in these published experiments, whereas females and a possible synergetic interaction between pheromones and carcass odour have not been investigated. In natural habitats, hide beetles feed on carrion or faecal pellets and males esterify ingested fatty acids to isopropyl esters in their pheromone glands [27]. Consequently, the production of the fatty acid isopropyl esters depends on the existence of an appropriate feeding substrate.

From a forensic entomological point of view, an important finding is that the arrival of adult hide beetles at the cadaver is predictable between 5 and 11 days after death in Hawaii (in mesophytic and xerophytic habitats) and other countries of the world [14]. Therefore, the presence of hide beetles can be used for the estimation of the post-mortem interval (PMI) in forensic investigations [30,31]. Forensic chemoecological studies have revealed that freshly emerged male hide beetles are attracted by the odour of piglet carcasses during the post-bloating stage as early as 9 days after death (T_mean_ = 27°C) and that benzyl butyrate is a key component for beetle attraction [9]. However, females are not attracted by the odour of piglet carcasses in various stages of decay [9]. Therefore, in our previous study [9], we have hypothesized that male pheromones, which are responsible for the above-mentioned mixed-sex aggregations [23], also might play a role in far-range female attraction when combined with cadaver odour.

As initially indicated, *D. maculatus* is a cadaver inhabitant that prefers a narrow succession niche in order to reduce competition with high blowfly activity [4]. Therefore, we expect that virgin female hide beetles are possibly attracted by a combination of cadaver odour and male pheromones. This guarantees reproductive possibilities because of the presence of receptive males on an appropriate substrate for feeding and oviposition.

Hence, the aim of the present study has been to investigate if a combination of cadaver odour and male sex pheromones is attractive to virgin female hide beetles. Furthermore, we have studied at which decomposition stage the first virgin females respond to the respective cadaver odour when combined with male sex pheromones.

## Methods

### Piglet cadavers

Five out of seven piglet cadavers (*Sus domesticus*) were used for the collection of headspace volatiles throughout the decomposition period. We used piglets for our study because they resemble human torsos in hair coverage, the ratio of fat to muscle, biochemistry and physiology [32,33]. Therefore, we expected a similar odour-profile both in quality and in relative amounts of the cadaveric volatile compounds. Despite the differences in carcass size and weight between piglet cadavers and human corpses, size is not significant in an insect-free decomposition process [34] in accordance with our experimental set-up. The piglets were exposed between April 1 and April 25, 2009 in plastic boxes (50 cm × 40 cm × 30 cm) at the Institute for Legal Medicine (University of Bonn, Germany) and covered with gauze to prevent the access of blowflies. The ambient temperature was recorded with an ebro EBI-6 data logger (ebro Electronic GmbH, Germany). The remaining two cadavers were in the dry-remains stage after having been exposed in an incubator at 25°C from the end of January until the beginning of April 2009. At night, the cadavers were positioned beside a radiator (T_mean_ = 26°C) in a closed room and, during the daytime, were placed outdoors on the roof of the Institute for Legal Medicine. The mean temperature of the radiator (T_mean_ = 26°C) was approximately the same as the mean temperature of the whole exposure period (T_mean_ = 27°C). Therefore, we excluded a possible temperature influence on carcass odour development because of the alternating exposure indoors and outdoors. We exposed the cadavers in plastic boxes and consequently avoided contact with different ground substrates on different exposure sites as a precaution for the development of a similar cadaveric odour profile.

### Headspace samples

Headspace volatile samples were collected between April 1 and April 25, 2009 from seven piglet carcasses at four defined decomposition stages: bloated stage (days 2–5 after death), post-bloating stage (days 5 – 12 after death), advanced decay (days 17 – 28 after death) and dry remains (days 71 – 91 after death). Two different piglets were used for each decomposition stage (except for advanced decay). For preparation of the headspace extracts, the adsorbed volatiles (in 5 mg Porapak® Q (Waters Division of Millipore, Milford, MA, USA)) were eluted with 3 × 50 μl pentane/acetone (9:1) (Sigma-Aldrich, Munich, Germany, HPLC grade) and, at the end of this procedure, we achieved an elution volume of approximately 80 μl. The sampling time was 4 h. The whole collection procedure was as described in detail in our previous study [9].

In the bioassays (see below), we used the following headspace samples, which were diluted 1:100 with pentane (Sigma-Aldrich, Munich, Germany, HPLC grade): bloated stage (day 3 post-mortem), post-bloating stage (days 9 and 10 post-mortem), advanced decay (day 22 post-mortem) and dry-remains stage (day 75 post-mortem). With such a high dilution of cadaver odour and/or the supply of only one male gland equivalent of (Z9)-unsaturated fatty acid isopropyl esters (see below), we simulated the natural situation of far-range beetle attraction in our small Y-olfactometer set-up.

### Rearing of hide beetles

*D. maculatus* was reared at the Institute of Experimental Ecology (University of Ulm, Germany) and used for gland extraction and bioassays. The breeding stock was kindly provided by Carsten Kopleck from the Alexander Koenig Zoological Museum (ZFMK, Bonn, Germany). The beetles were reared over several generations in plastic boxes (30 cm × 30 cm × 15 cm) on cat food (IAMS Kitten, http://www.1a-zoo.de) under a light regime of 12:12 light/dark at a temperature of 28°C and a humidity of 80%. From time to time, mouse carrion was added to avoid possible changes in the responsiveness to carcass odour because of the artificial breeding conditions. Therefore, with such a temporary carcass supply, we attempted to maintain the ecological comparability of museum stock beetles with their free-living relatives. Water was provided ad libitum by using moistened cotton pads. The cat food was placed into the boxes at a coating thickness of 3 cm and also served as a rearing substrate. Pupae were removed from the rearing substrate and subsequently reared until eclosion in plastic boxes furnished with dry tissue paper.

Newly eclosed males and females were separated immediately after emergence and kept isolated. Females were tested in the bioassay at 2 to 3 weeks after eclosion, when they were receptive, and males were used after the same time span for gland extraction. For bioassays with newly emerged females, beetles were tested 24 hrs after eclosion.

### Gland extracts

For gland extraction, 2- to 3-week-old unmated males with fully developed pheromone glands and gonads were used [28]. The abdominal segments 4 and 5 were dissected with a razor blade after the beetles had been cooled for 30 min at −25°C in a freezer. Subsequently, the abdominal segments of 20 beetles were extracted in 1 ml pentane (Sigma-Aldrich, Munich, Germany, HPLC grade) for 24 hrs at room temperature and, afterwards, the pentane was evaporated to 400 μl under a gentle stream of nitrogen. Aliquots of 20 μl, representing one gland equivalent (i.e. about 1 μg of fatty acid isopropyl esters [25]), were used for each test in the bioassay (see below).

### Synthetic reference chemicals of cadaver volatiles and pheromones

In order to test synthetic analogues of cadaver odour and/or (*Z*9)-unsaturated fatty acid isopropyl esters in the Y-olfactometer bioassays (see below), we produced the following mixtures:

A synthetic cadaver odour containing 11 electrophysiologically active carcass volatiles was produced according to the chemical analyses of headspace samples of a piglet cadaver in the post-bloating stage (day 9 post mortem) reported in our previous study [9]. Of this cadaver odour mix containing isoamyl butyrate (20 ng), ethyl butyrate (3 ng), hexyl butyrate (1 ng), 1-octen-3-ol (4 ng), propionic acid (20 ng), 2-methyl butyric acid (20 ng), 3-methyl butyric acid (20 ng), benzyl butyrate (0.2 ng), butyramide (0.5 ng), 2-phenylethanol (8 ng) and 2-phenylethyl butyrate (1 ng), 20 μl were used for each test in the bioassay (see below).

A synthetic mixture of synthetic (*Z*9)-unsaturated fatty acid isopropyl esters was produced according to the amounts of one male gland equivalent [25]. These amounts elicited maximal attraction of male hide beetles [28]. Of this pheromone mixture containing isopropyl (*Z*9)-dodecenoate (0.1 ng), isopropyl (*Z*9)-tetradecenoate (150 ng) and isopropyl (*Z*9)-hexadecenoate (200 ng), 20 μl were used for each replicate in the bioassay (see below). Isopropyl (*Z*9)-dodecenoate and isopropyl (*Z*9)-tetradecenoate were kindly provided by Frans Griepink (*PHERO*BANK, Wageningen, The Netherlands). Isopropyl (*Z*9)-hexadecenoate was synthesized by estrification of (*Z*9)-hexadecenoic acid with 2-propanol by using 4-dimethylamino pyridine as a catalyst and N,N’-dicyclohexyl carbodiimide as a condensation reactant [35].

### Bioassays

The attractiveness of natural and synthetic pheromones and carcass volatiles, respectively (Table 2), was tested in a Y-olfactometer. The olfactometer consisted of a block of plexiglass (9 cm x 7 cm x 1 cm) into which a Y-shaped duct system (0.5 cm deep) had been milled (arm length 2.5 cm, stem length 2.5 cm). The Y-shaped duct system was connected to a lockable cylindric plexiglass arena (diameter 4 cm) via which the beetles could enter the stem of the ‘Y’. The olfactometer was placed on a sheet of paper (DIN-A4) after this had been impregnated with the volatile solution to be tested (test arm) and an equal amount of the pure solvent (control arm), respectively. Liquids were applied to each arm at a distance of 0.5 cm from the crossing point of the ‘Y’ by using a micro-syringe (100 μl, Göhler HPLC-Analysentechnik, Chemnitz, Germany) and the solvent was allowed to evaporate for 30 s before the olfactometer was placed on the paper sheet. Beetles were placed singly into the cylindric plexiglass arena and, after a settling time of 1 min, the exit of the arena was opened. A choice was recorded if a beetle entered one of the arms of the Y-olfactometer and passed an imaginary line 1 cm after the branching point. If a beetle did not make a decision within an observation time of 3 min, it was discarded from the experiment and substituted by another individual to maintain an equal sample size. Only one of twenty beetles (5%) had to be discarded from any particular experiment. In order to eliminate possible side effects, test and control arms were regularly changed. Each hide beetle was tested only once (N = 20 for each test) and, after every test, the olfactometer was cleaned with pure ethanol (Merck, Darmstadt, absolute for analysis). We performed all behavioural tests in a climate chamber under red light at 28°C and a relative humidity of 80%.

**Table 2:** Compilation of all conducted test conditions with statistical results in a Y-olfactometer bioassay

**Sample**	**Control**	**n**	**P**	**Beetles tested**
gland extract^a^	Pentane	20	0.132	virgin females^i^
synthetic				
pheromones^b^	Pentane	20	0.588	virgin females^i^
bloated^c^				
+ pheromones^b^	Pentane	20	0.412	virgin females^i^
				newly emerged
post-bloated^d^	Pentane	20	0.588	females^j^
post-bloated^d^	Pentane	20	0.412	virgin females^i^
post-bloated^d^				
+ pheromones^b^	Pentane	20	0.058	virgin females^i^
synthetic copy				
of post-bloated^g^				
+ pheromones^b^	Pentane	20	0.058	virgin females^i^
advanced decay^e^				
+ pheromones^b^	Pentane	20	0.412	virgin females^i^
				newly emerged
dry remains^f^	Pentane	20	0.588	females^j^
dry remains^f^	Pentane	20	0.588	virgin females^i^
dry remains^f^				
+ gland extract^a^	Pentane	20	< 0.001	virgin females^i^
dry remains^f^				
+ pheromones^b^	Pentane	20	0.058	virgin females^i^
benzyl butyrate^h^				
+ pheromones^b^	Pentane	20	0.588	virgin females^i^

^a^ pentane extract of a male gland = 1 gland equivalent.

^b^ synthetic (Z9)-unsaturated fatty acid isopropyl ester bouquet (1 gland equivalent).

^c^ headspace sample day 3 post-mortem.

^d^ headspace sample day 10 post-mortem.

^e^ headspace sample day 22 post-mortem.

^f^ headspace sample day 75 post-mortem.

^g^ 11 electrophysiologically active compounds in the post-bloating stage (day 9 post-mortem).

^h^ same concentration of the electrophysiologically active compound benzyl butyrate as that in the post-bloating stage (day 9 post-mortem).

^i^ 2–3 weeks old.

^j^ 24 hrs old.

Choice experiments were analysed by the one-sided binomial probability test. Sample sizes (n) and significance levels (P-value) are given for each test.

### Statistics

Choice experiments were analysed by the one-sided binomial probability test and between different conducted binomial probability tests by the 2 x 2 Chi^2^-test with a P-value correction according to Benjamini and Hochberg (P <0.05), in all cases with the use of SPSS (Version 13.0, SPSS GmbH Software, Germany). We computed a one-sided test [36], because a priori we expected that cadaver odour and/or male sex pheromones would have no repellent effect against virgin female hide beetles and that consequently their response to cadaver odour and/or male sex pheromones would always be equal or greater than that to the solvent control.

## Results

**Figure 1:**
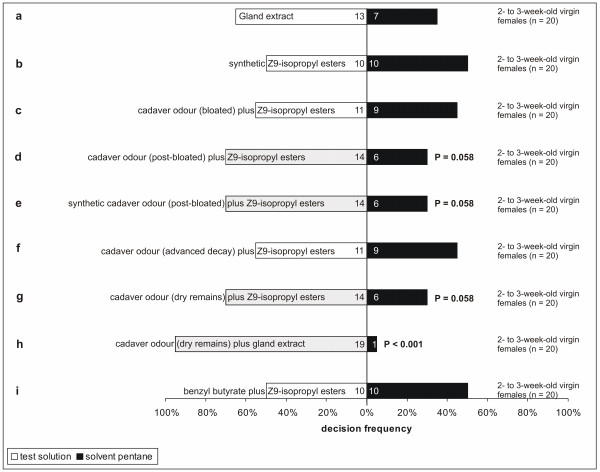
**Responses of female hide beetles in Y-olfactometer choice experiments to male gland extract, synthetic (Z9)-unsaturated fatty acid isopropyl esters, benzyl butyrate plus (Z9)-unsaturated fatty acid isopropyl esters and different cadaver odours (bloated, post-bloated, synthetic copy of post-bloated, advanced decay, dry remains) either plus (Z9)-unsaturated fatty acid isopropyl esters or plus male gland extract (one-sided binomial probability tests) versus pure solvent control.** Those test solutions which elicited significant results (P <0.001) or strong tendencies (P = 0.058) are highlighted in greyish colour. Numbers inside of the bars denote identified ratios of the particular test preferences. Distinct letters label different test series. Bloated stage: day 3 post-mortem; Post-bloating stage: day 10 post-mortem; Mimicked post-bloating stage: day 9 post-mortem; Advanced decay stage: day 22 post-mortem; Dry-remains stage: day 75 post-mortem.

**Figure 2:**
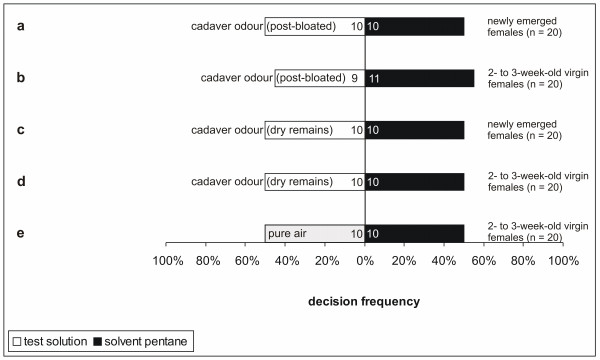
**Responses of female hide beetles in Y-olfactometer choice experiments to cadaver odour (post-bloated or dry remains) and to pure air (one-sided binomial probability tests) versus pure solvent control.** Numbers inside of the bars denote identified ratios of the particular test preferences. Distinct letters label different test series. Post-bloating stage: day 10 post-mortem; Dry-remains stage: day 75 post-mortem.

A synthetic mixture of (Z9)-unsaturated fatty acid isopropyl esters was not more attractive for virgin female hide beetles than the solvent control, whereas the whole male gland extract showed a weak tendency to be more attractive than the pure solvent (Table 2 and Figure 1). Combinations of cadaver odour (post-bloated, synthetic copy of post-bloated and dry remains) with synthetic (*Z*9)-unsaturated fatty acid isopropyl esters appeared to be more attractive (not significant but with a strong tendency) for receptive female hide beetles than the solvent controls (Table 2 and Figure 1). Combinations of (*Z*9)-unsaturated fatty acid isopropyl esters, either with the odour of bloated piglet cadavers or with the odour of cadavers in the advanced decay stage showed the same attractiveness than the solvent control (Table 2 and Figure 1). Only the combination of cadaver odour of a piglet in the dry remains stage with one whole male gland extract was significantly more attractive for receptive female hide beetles than the solvent control (Table 2 and Figure 1). Additionally, this combination was significantly more attractive than the gland extract alone (2 × 2 Chi-square test; Chi^2^ = 5.738; d.f. = 1; P = 0.016, Figure 1, test series a and h) and also significantly more attractive than the dry remains odour alone (2 x 2 Chi-square test; Chi^2^ = 10.395; d.f. = 1; P = 0.001, Figure 1 and Figure 2, test series h (Figure 1) and d (Figure 2)). Concerning the constitution of male sex pheromones, we furthermore detected a significant difference in beetle attraction to dry-remains odour when providing an additional male gland extract compared with additional synthetic (*Z*9)-unsaturated fatty acid isopropyl esters (2 × 2 Chi-square test; Chi^2^ = 4.408; d.f. = 1; P = 0.035, Figure 1, test series h and g). Cadaver odour without male sex pheromones of a decomposed piglet in the post-bloated stage and volatiles released by carcasses in the dry-remains stage seemed to be neither attractive for newly emerged nor receptive females (Table 2 and Figure 2). The combination of benzyl butyrate and (*Z*9)-unsaturated fatty acid isopropyl esters had no influence on the behaviour of virgin female hide beetles (Table 2 and Figure 1).

## Discussion

### Are virgin female hide beetles attracted by a combination of cadaver odour and male sex pheromones?

Our results indicate that cadaver odour alone [9] is possibly not sufficient for the attraction of 2- to 3-week-old virgin female hide beetles. One male gland extract with all sex pheromone constituents shows a non-significant but slight tendency for an attraction effect. Interestingly, in our Y-olfactometer tests, the odour combination of a piglet in the dry remains stage and one male gland extract was more attractive than the respective single odour bouquet or gland extract, respectively. We hypothesize that this behaviour is adaptive because virgin females that are willing to reproduce are guided to both mates and oviposition substrates ensuring immediate reproductive possibilities with minimal energetic costs. This is in accordance with the mating system of the old-house borer *Hylotrupes bajulus* in which males also arrive first at oviposition sites (pinewood) and virgin females are attracted by a combination of pine wood odour and the male sex pheromone [37].

In bark beetles and many other herbivores, females responding to male-derived aggregation pheromones are also guided to feeding and oviposition sites, because these pheromones are typically released after males have located host plants. For instance, males of the boll weevil *Anthonomus grandis* and the sunflower seed weevil *Smicronyx fulvus* settle on their hosts before the respective females become active [38,39].

However, the question remains as to why young virgin female hide beetles are not attracted to carrion odour itself in order to have access to food. We cannot exclude that they feed on alternative animal food sources other than carcasses. A further explanation of our finding that newly emerged (and thus starved) females are not attracted to carrion odour itself (Figure 2) could be the importance of other, but up to now unknown, cues (such as visual cues) that we have not tested in our experiments.

Our results indicate that the attractiveness of the pheromone is linked to the presence of *D. maculatus* males on an appropriate substrate. In former investigations, male hide beetles have been shown to have to feed on fatty acids as precursors for the production of isopropyl esters. This is stimulated by the oleic acid present in the food substrate or in the faecal pellets of the beetles themselves [27]. Concerning carcass partitioning, *D. maculatus* has been established to have strict feeding preferences for cadaver skin and moist fleshy or ligamentous tissue [4]. Furthermore, newly emerged males are known to arrive at a carcass exactly in the post-bloating stage, the stage at which the relative proportion of the key attractant benzyl butyrate is at a maximium [9]. For this reason, we consider the hide beetle to be a highly specialized cadaver inhabitant and, thus, it might be important for virgin females to synchronize their reproduction in accordance with the availability of feeding and breeding substrates [40], as indicated by a combination of cadaver odour and male sex pheromones.

In our bioassay, we have found that young virgin female hide beetles respond more strongly to cadaver odour (dry remains) in combination with natural rather than with synthetic male pheromone compounds (Figure 1). We can explain this difference on the basis of the missing pheromone compounds in our synthetic mixture in comparison with the higher diversity of esters in the natural pheromone bouquet, which has been shown to consist of 11 components [25], whereas our synthetic mixture contains only 3 compounds. Our data suggest, on the other hand, that only three electrophysiologically active (*Z*9)-unsaturated fatty acid isopropyl esters possibly provide an important contribution to the signal that attracts young virgin females when offered in combination with cadaver odour of dried-out piglet cadavers. Additionally, by conducting all except one of our bioassays with synthetic (Z9)-unsaturated fatty acid isopropyl esters instead of gland extracts (Figure 1), we have excluded the possibility that non-pheromone compounds, which are ever-present in extracts of complete abdominal segments, might have an unwanted behaviour-modifying effect. We regard the recognition of the dried-out decomposition stage in combination with sexually mature and pheromone-emitting males [28] as being essential for virgin females, because this dried substrate (cadaver skin) is the preferred food resource for the larvae [4].

### During which decomposition stage do the first virgin females respond to cadaver odour when combined with male sex pheromones?

The determination of the post-mortem interval of badly decomposed human remains is an important question in medicolegal investigations [5]. The succession of cadaver-associated insects can be used to address this question, because many of them are attracted to specific decomposition stages, probably guided by stage-specific carcass volatile bouquets [9,10].

Our results show a tendency that the at the cadaver arriving young virgin females of *D. maculatus* possibly respond to cadavers with regard to a combination of their respective odours and the synthetic male pheromones as early as the post-bloating stage (10 days after death; T_mean_ = 27°C). The odour of a post-bloated piglet cadaver alone seems to be attractive neither for freshly emerged nor for 2- to 3-week-old virgin females (Figure 2) nor the odour of bloated piglet cadavers [9]. Our data provide a careful hint that the prior arrival of males (guided by benzyl butyrate) could be essential for young virgin females to settle on a corpse at this early time. A previous study has reported the presence of adult dermestid beetles as early as 3 to 5 days and 10 days post-mortem, respectively, but no reports of infestation with dermestid larvae at this early stage are available [41]. Moreover, the presence of adult hide beetles has been reported as early as 4 days post-mortem [42]. Our previous work [9] supports these observations for an arrival time of male hide beetles between day 9 and 10 post-mortem but not for arrival times as early as 3 to 5 days post-mortem. The odour of a bloated piglet cadaver (day 3 post mortem) is not more attractive than the control, neither for freshly emerged males nor for 2- to 3-week-old males with differentiated glands and gonads [9]. However, our results suggest that male hide beetles arrive even earlier than 9 days post-mortem, because time is required for pheromone gland differentiation and precursor uptake before they can attract receptive females [27]. In order to verify this suggestion, we plan to collect and sex early arriving hide beetles on carcasses in the field and will also determine the length of time that the males stay at the cadaver before maturing and calling.

The earliest record of dermestid larvae on a human corpse was reported at 21 days post-mortem in the early stages of advanced decay and more larvae were collected after 43 days [8]. Entomological investigations in the course of a real criminal case have reported adults, pupae and larvae of the hide beetle within a time range of 3 to 6 months [43]. After mating, female hide beetles can produce fertile eggs for the rest of their life, i.e. approximately 3 months [44]. This could be a general problem for a precise estimation of the PMI after hide beetle infestation, because oviposition at a later time point after the mating event is generally possible.

By means of a completely synthetic test condition consisting of a mixture of synthetic cadaver odour (post-bloated stage) and synthetic (Z9)-unsaturated fatty acid isopropyl esters, we tendentially attracted young virgin female hide beetles in our Y-olfactometer bioassay. In contrast to the completely synthetic cadaver odour bouquet, benzyl butyrate alone in combination with (Z9)-unsaturated fatty acid isopropyl esters seemed not to be sufficient to attract young virgin female hide beetles. Obviously, the butyric acid ester alone is sufficient to attract freshly emerged male hide beetles [9]; however, it appears that young virgin females need one or more additional electrophysiologically active compounds of the cadaver odour bouquet in combination with, or instead of, benzyl butyrate plus male sex pheromones in order to detect an appropriate substrate for mating and oviposition.

We avoid speculation about complex and only tendential differences in young virgin female hide beetle attraction between the remaining bioassay results (either with odour of bloated piglet cadavers in combination with synthetic pheromones or with odour of piglet cadavers in advanced decay in combination with synthetic pheromones) and the already above discussed results. Due to a limit of available headspace odour samples we were not able to increase sample sizes to address this problem.

## Conclusions

The present study is the first to provide indications that the attraction of young virgin female hide beetles to cadavers can be mediated by a mixture of carcass volatiles and male sex pheromones. Cadaver odour alone, as sufficient to attract newly emerged males, appears not to be sufficient to attract 2- to 3-week-old virgin female hide beetles. Newly emerged males might respond to cadaver odour before the reaction of young virgin females and thus indicate them as an appropriate site for feeding, mating and oviposition by releasing pheromone in the presence of the substrate.

The finding (even if only tendentially) that the first young virgin females seem to respond to cadaver odour in combination with male sex pheromones as early as the post-bloating stage (10 days after death; T_mean_ = 27°C) might be of general importance for forensic entomologists, although one should keep in mind that our results are only based upon a specific age group of young virgin females. Furthermore, we have used piglet cadavers in our study and not human corpses. We cannot exclude the possibility that there is a faster or slower change in the odour profile of human carcasses, which in turn might influence the time frame of the appearance of young virgin female hide beetles. Nevertheless, our results suggest that the retrieval of hide beetles or hide beetle remains from cadavers is not necessarily an indication for extended post-mortem intervals. However, this possibility is based on laboratory experiments with the decomposition odour of piglet cadavers only and needs to be confirmed under field conditions. Finally, one should keep in mind that all our published results and the conclusions of this study are based on a relatively high mean temperature (T_mean_ = 27°C). Therefore, we have cited only studies carried out under similar temperature conditions in order to make our results and conclusions comparable. In forensic entomological case work, the application of data of insect developmental times and/or decay rates of cadavers in crime-scene investigations only with comparable conditions of the ever-present abiotic parameters such as temperature and humidity is essential. For instance, relatively high temperatures as a prerequisite for optimal hide beetle development can be recorded in death scenes inside human habitation when the central heating is turned up to its highest degree and all windows are closed, independent of the geographic region [45].

## Competing interests

The authors declare that they have no competing interests.

## Authors’ contributions

CvH carried out the research, data analysis and drafted the manuscript. JR carried out the chemical synthesis of Isopropyl (*Z*9)-hexadecenoate and helped to draft the manuscript. MA participated in the study design and coordination and helped to draft the manuscript. All authors read and approved the final manuscript.
